# Using a spatial autoregressive model with spatial autoregressive disturbances to investigate origin-destination trip flows

**DOI:** 10.1371/journal.pone.0305932

**Published:** 2024-06-26

**Authors:** Linglin Ni, Dapeng Zhang

**Affiliations:** 1 Logistic School, Beijing Wuzi University, Beijing, China; 2 School of Public Administration and Policy, Renmin University of China, Beijing, China; University of Bologna, ITALY

## Abstract

Spatial interaction models with spatial origin-destination (OD) filters are powerful tools to characterize trip flows in space, which is a classic and important problem in regional science. To the authors’ knowledge, existing studies adopting OD filters mostly specify the spatial dependence as an autoregressive process, which may not be the full picture of spatial effects. To examine the problem, this paper proposes the hypotheses that 1) spatial OD dependences can take place in both the spatial autoregressive term and the spatial error term in a spatial interaction model. 2) Estimating a spatial autoregressive model with spatial autoregressive disturbances (SARAR) model with OD filters would disentangle where the spatial dependence exists and by how much. 3) The marginal effects obtained from SARAR models would be preferred to analysts when SARAR models outperform spatial autoregressive (SAR) models and spatial error models (SEM) from the statistical point of view. To assess these hypotheses, this paper specifies, estimates, and applies SARAR models with OD filters to investigate trip distributions. By comparing against alternative models, this paper investigates the estimation results in SAR, SEM and SARAR models using an empirical data collected from Hangzhou, China. The contribution of this paper is to be the first in developing an SARAR model with OD filters for trip distribution analyses and examining its performance.

## 1. Introduction

Spatial interaction models with spatial filters on both the origin and the destination (OD) have become a popular way to study OD trip flows, because researchers believe spatial dependence at the flows’ starting points and ending points both affect flow patterns. The spatial OD filters can characterize the influence due to spatial proximity of nearby zones on a traffic analysis zone serving as both an origin and a destination. In a spatial interaction model, OD filters can be specified as spatial autoregressive terms in the dependent variable (SAR) or in the error term (SEM). Specifically, the SAR specification can have three spatially lagged terms to capture spatial dependences at trips’ origins, trips’ destinations, and the interactions of trips’ origins and destinations [1]. In the SEM specification, the spatial effect can be captured by three similar spatially lagged terms, but they are specified in the error terms of the econometric equation.

Selections of the best spatial econometric model specification usually start with investigating one SAR model and one SEM [1]. According to the literature [2], both SAR and SEM specifications can capture spatial dependences effectively. However, the underlying philosophies differ: SAR models assume that spatial dependence occurs within the dependent variable, without directly specifying spatial effects in the attributes or the unexplained portion of the dependent variable. On the other hand, SEM specifications assume that the spatial dependences takes place in the un-explanatory part of the dependent variable, rather than the dependent variable itself or the attributes. Both philosophies can somewhat explain the spatial effects in traffic flow: on the one hand, specifying spatial dependences in the dependent variable, i.e., SAR specifications, implies that traffic flows perform similarly when the origin and/or the destination are close to each other. On the other hand, specifying spatial dependencies in the error term, i.e., SEM specifications, implies that a shock in traffic flows impacts not only the immediate area but also neighboring error terms. This process, in turn, indirectly propagates to other flows.

Not only the underlying philosophies are different between SAR and SEM specifications, but the estimated marginal effects also differ. When the absolute values of spatial coefficients are close to 1, the difference in marginal effects of explanatory variables is distinguishable. That is because derivatives of the traffic flow on explanatory variables would involve the spatial lag terms in the SAR specification, which creates a spillover effect across traffic flows. Nonetheless, marginal effects in the SEM would simply be the coefficients of explanatory variables. The difference in marginal effects would drive different policy implications in transportation planning and management. For example, the accessibility of transportation services usually presents a positive effect on the traffic flow. Public agencies can adjust accessibility policies to intervene traffic flow of certain areas. The amount or intensity of policy intervention relies on the degree to which marginal effects correspond to varied social and economic investments.

Given the differences in model specifications, philosophies, and interpretations, the following hypothesis is proposed: spatial dependences in the OD flow can take place in both the spatial autoregressive term and the spatial error term depending on the real-world scenario. Testing the hypothesis can be executed by specifying and estimating a spatial autoregressive model with spatial autoregressive disturbances (SARAR) model with OD filters. The SARAR with OD filters, which has been theoretically specified [1] but not estimated or applied in the empirical studies, can estimate the spatial dependences on the dependent variable and the error term simultaneously. The estimated spatial coefficients can reveal where the spatial dependence exists and by how much. While the marginal effects derived from the SARAR specification are theoretically similar to those of the SAR model, analysts may prefer the SARAR approach when it demonstrates superior statistical performance over the SAR and SEM specifications.”

Hence, this paper estimates a series of spatial econometric models with OD filters including the SAR, the SEM, and the SARAR specifications to disentangle spatial dependences in the OD travel flow. The detailed estimation methods are proposed and applied on an empirical trip distribution data in Hangzhou, China, which contains the OD travel flow matrices, socio-economic factors, and transportation infrastructure measures. Moreover, OD flow matrices at four separate times have been collected: Thursday morning, Sunday morning, Thursday afternoon and Sunday afternoon. This paper compares the performance of all specifications with OD filters to gain additional insights into marginal effects at separate times.

This paper aims to enrich the existing literature by estimating and applying the SARAR specification with OD filters. Meanwhile, this paper is the pioneer study to examine the philosophies of the SAR, the SEM, and the SARAR specifications and how the difference in spatial dependences drives the variation in marginal effects of explanatory variables. Finally, this paper deepens the understanding of spatial dependences in trip distribution at different times of day and days of week.

The next section will review the literature of the SARAR specification in OD flow analyses. Then, the model specification and estimation methodology are introduced in Section 3. Section 4 conducts an empirical analysis using the SARAR specification, compares its performance with alternative models, and discusses policy implications. Section 5 concludes the paper with discussions and the next steps.

## 2. Literature review

Spatial econometric models with OD filtering, proposed in LeSage and Pace [1], have become a popular method in regional science, as it can capture spatial dependences at both origins and destinations. By calculating explanatory variables’ marginal effects, analysts can reveal the origin effects, destination effects and total effects of the change in an explanatory variable of a certain region on the flow pattern arising from spatial effects of origins and destinations [3]. Several empirical studies have been conducted borrowing the OD filtering specifications, but all of them, to the best of knowledge, adopted SAR specifications, except LeSage and Fischer explained OD filtering on explanatory variables [1]. For example, Margaretic et al. [4] analyzed the spatial dependence in air passenger flows with SAR specifications. LeSage and Llano [5] used the Bayesian Markov Chain Monte Carlo method to analyze commodity flows of 18 Spanish regions by the SAR specification. Ni et al. [6] used the SAR specification to investigate less-than-truckload flow across all provinces in China. Ni et al. [7] also used the SAR specification to analyze the weekly average travel flow via the 51 zones in Hangzhou, China. Note that the empirical data in last mentioned literature used weekly data. This paper uses a recently obtained daily travel data, which means this paper can explore the difference in trip distribution across weekdays and weekends, as well as mornings and afternoons. The main reason for the existing literature to prefer SAR specifications may be that spillover effects in SAR can be incorporated in the interpretation of marginal effects, adding color in characterizing explanatory variables’ effects on travel flow.

Nonetheless, SAR specification can only capture part of the spatial effect, and a natural extension of SAR is to add spatial terms in the disturbance terms, resulting in the so-called SARAR models. SARAR can address the two most important spatial effects simultaneously: spatial dependence and spatial heterogeneity, specifically referring to heteroskedasticity in the context of spatial dependence, as elucidated by Anselin [8]. Empirical studies usually found that SARAR performs better among SAR, SEM and SARAR. For example, Li et al. [9] adopted a 3-D SARAR hedonic model to unveil the impact of polluted river on the high-rise apartment market of Guangzhou. They claimed the spatial dependence in the error term after fitting a SAR cannot be ignored. In COVID prevalence analysis conducted by Sun et al. [10], SARAR outperformed the linear model, SAR model, and SEM in explaining the spreading over U.S. counties. Hence, in OD filtering analysis, analysts should leverage SARAR models so that spatial effects can be examined in a more comprehensive way and lead to a more trustworthy interpretation of marginal effects with respect to spatial processes, which is one of the key objectives of this paper.

Trip distribution is one of the most important and conventional problems in transportation research [11]. It explains where a trip starts and ends. Understanding trip distribution is a pre-requisite work in solving various transportation problems. OD matrix balancing techniques, gravity models, and spatial interaction models are usually used in practice to calibrate trip distribution. The challenge of trip distribution analysis before the big data era was data collection. Analysts have to rely on somewhat manual traffic counts or travel survey to extract a sample OD matrix, and the accuracy is often in doubt [12]. With the development of intelligent transportation technologies, analysts can access real time trip distribution with full population of data. Then, the research focus extends from OD matrix calibration to trip distribution explanation, i.e., establishing the relationship between explanatory variables and trip distribution. By having these relationship, urban administration officers can intervene traffic flow by adjusting explanatory variables to address traffic problems. However, trip distributions are more sophisticated than what explanatory variables can explain. Spatial dependence, spatial heterogeneity, and other error reduction schemes are needed to improve the performance of trip distribution models. However, such studies are far from completion. Hence, this paper, in the context of leveraging big data to model trip distributions, bridges the gap of the need in trip distribution modeling and the current state of art in spatial interaction models by fitting OD filtering SARAR models on the trip distribution data collected in Hangzhou, China.

## 3. Methodology

SARAR models with OD filters can jointly examine spatial dependences in the dependent variable and the error term. One of the objectives in this paper is to specify, estimate and apply SARAR models. It’s worth to note that spatial filters can be also specified on the explanatory variable terms, which enables the so-call SLX (spatially lagged X) model [13]. The SLX specification can be combined with SAR and SEM specifications to establish a series of alternative specifications. Based on the relevant literature, the SLX specification may present advantages in the model selection from the mathematical point of view. However, in the trip distribution context, the main focus is often on the spatial dependence of the dependent variable and the error structure. Specifically, OD flow itself is an important variable in transportation analyses, and exploring its spatial dependence is of the greatest interest. In addition, analysts often observe a high variation in the OD matrix, meaning looking into the disturbance term can help improve the overall model performance. This paper investigates SAR, SEM, and SARAR specifications, which is also consistent with Anselin’s point of view in that spatial dependence and spatial heterogeneity are the two most important specifications in the spatial data analysis [2].

Assume **y** denotes the traffic flow between a set of origins *k*(1⋯*K*) and a set of destinations *l*(1⋯*L*). Let *N* = *K*×*L*, the size of **y** is (*N*×1).


$$y=\left[\begin{array}{ccc} y_{11} & \cdots & y_{1k}\\ \vdots & \ddots & \vdots \\ y_{L1} & \cdots & y_{KL} \end{array}\right]$$ (1)

The SARAR model specification can take the form

$$y=\rho_{o}W_{o}y+\rho_{d}W_{d}y+\rho_{w}W_{w}y+X_{o}\beta_{o}+X_{d}\beta_{d}+X_{w}\beta_{w}+\beta_{c}+\varepsilon$$

$$\varepsilon =\lambda_{o}W_{o}\varepsilon +\lambda_{d}W_{d}\varepsilon +\lambda_{w}W_{w}\varepsilon +\mu$$

$$\mu ∼N(0,\sigma^{2}I_{N})$$

$$\rho_{w}=-\rho_{o}\rho_{d}$$

$$\lambda_{w}=-\lambda_{o}\lambda_{d}$$ (2)

where ***W***_***o***_ **= *W*⨂*I***_***N***_ is the spatial matrix capturing the spatial lag arising from the origin of traffic flows. The operator ⨂ denotes the Kronecker product. ***I***_***N***_ denotes the unit matrix of size (*N*×*N*) with 1 on its diagonal. The same idea applies to ***W***_***d***_ **= *I***_***N***_**⨂*W*** which captures the spatial dependence at the destination of traffic flows. The spatial matrix ***W***_***w***_ **= *W*⨂*W*** is used to indicate the interaction of the origin and destination dependence. The spatial matrices ***W***_***o***_**,*W***_***d***_, and ***W***_***w***_ are employed to define spatial proximity in autoregressive terms. The parameter ***ρ***{*ρ*_*o*_,*ρ*_*d*_,*ρ*_*w*_} capture the spatial dependencies at origin, destination, and their interaction on the dependent variable, while ***λ***{*λ*_*o*_,*λ*_*d*_,*λ*_*w*_} measures the intensity of spatial dependence in the disturbance term.

As pointed out by LeSage and Pace [1], the constraints *ρ*_*w*_ = −*ρ*_*o*_*ρ*_*d*_ and *λ*_*w*_ = −*λ*_*o*_*λ*_*d*_ of the model can be regarded to reflect the filter

$$(I_{N}-\rho_{d}W_{d})(I_{N}-\rho_{o}W_{o})=I_{N}-\rho_{d}W_{d}-\rho_{o}W_{o}+\rho_{d}\rho_{o}W_{w}$$

$$(I_{N}-\lambda_{d}W_{d})(I_{N}-\lambda_{o}W_{o})=I_{N}-\lambda_{d}W_{d}-\lambda_{o}W_{o}+\lambda_{d}\lambda_{o}W_{w}$$

This paper consistently applies these constraints, ensuring that *ρ*_*w*_ and *λ*_*w*_ are treated as known coefficients to enhance the tractability of the estimation process. This approach simplifies the model while providing a more manageable estimation process.

The explanatory variable ***X***{***X***_***o***_**,*X***_***d***_**,*X***_***w***_} captures the factors of origins, destinations, and their interaction of origins and destinations such as the distance between origins and destinations. The corresponding coefficients as well as the coefficients of constant are ***β***{***β***_***o***_**,*β***_***d***_**,*β***_***w***_,*β*_*c*_}, which can be used for deriving the marginal effects of explanatory variables.

The disturbance term ***ε*** includes three spatially lagged terms. The term *μ* is the idiosyncratic term with mean 0 and variance *σ*^2^.

When ***ρ***{*ρ*_*o*_,*ρ*_*d*_,*ρ*_*w*_} are not zero while ***λ***{*λ*_*o*_,*λ*_*d*_,*λ*_*w*_} are zeros, the SARAR model specification is reduced to a SAR model, which takes the form

$$y=\rho_{o}W_{o}y+\rho_{d}W_{d}y+\rho_{w}W_{w}y+X_{o}\beta_{o}+X_{d}\beta_{d}+X_{w}\beta_{w}+\beta_{c}+\varepsilon$$

$$\varepsilon ∼N(0,\sigma^{2}I_{N})$$

$$\rho_{w}=-\rho_{o}\rho_{d}$$ (3)

When ***ρ***{*ρ*_*o*_,*ρ*_*d*_,*ρ*_*w*_} are zeros while ***λ***{*λ*_*o*_,*λ*_*d*_,*λ*_*w*_} are not zero, the SARAR model specification becomes a SEM model, which takes the form

$$y=X_{o}\beta_{o}+X_{d}\beta_{d}+X_{w}\beta_{w}+\beta_{c}+\varepsilon$$

$$\varepsilon =\lambda_{o}W_{o}\varepsilon +\lambda_{d}W_{d}\varepsilon +\lambda_{w}W_{w}\varepsilon +\mu$$

$$\mu ∼N(0,\sigma^{2}I_{N})$$

$$\lambda_{w}=-\lambda_{o}\lambda_{d}$$ (4)

While these two reduced models can both capture the spatial effects rooting in the trip distribution data, the estimated marginal effects may vary dramatically. Let ***A*** = (***I***_***N***_−*ρ*_*o*_***W***_***o***_−*ρ*_*d*_***W***_***d***_−*ρ*_*w*_***W***_***w***_), the SAR model can be written as

$$y=A^{-1}X_{o}\beta_{o}+A^{-1}X_{d}\beta_{d}+A^{-1}X_{w}\beta_{w}+A^{-1}\beta_{c}+A^{-1}\varepsilon$$ (5)

In other words, the derivative of the traffic flow on explanatory variables would involve a spatial filtering process, which captures the spatial spillover effects. For example, ***X***_***o***_’s marginal effect is

$$SAR.ME(X_{o})=\frac{\partial y}{\partial X_{o}}=A^{-1}\beta_{o}$$ (6)

Let ***B*** = (***I***_***N***_−*λ*_*o*_***W***_***o***_−*λ*_*d*_***W***_***d***_−*λ*_*w*_***W***_***w***_), the SEM model can be written as

$$y=X_{o}\beta_{o}+X_{d}\beta_{d}+X_{w}\beta_{w}+\beta_{c}+B^{-1}\mu$$ (7)

where ***β***{*β*_*o*_,*β*_*d*_,*β*_*w*_} are the marginal effects which does not take any spatial process.


$$SEM.ME(X_{o})=\frac{\partial y}{\partial X_{o}}=\beta_{o}$$ (8)

Assuming two model specifications with similar estimated values of ***ρ***{*ρ*_*o*_,*ρ*_*d*_,*ρ*_*w*_} and ***λ***{*λ*_*o*_,*λ*_*d*_,*λ*_*w*_}, it indicates the intensity of spatial dependence in both specifications are similar, but the corresponding marginal effects may be vastly different. To obtain more reliable marginal effects in empirical studies, one way is to estimate all spatial coefficients in the SARAR model, which can locate spatial effects and derive the values of marginal effects.

This paper adopts a maximum likelihood estimation method to derive the coefficients in the SARAR model. The log-likelihood function takes the form

$$LL=-\frac{N}{2}\log\left(2\pi \sigma^{2}\right)+log|A|+log|B|-\frac{1}{2\sigma^{2}}{\left(ABy-BX_{o}\beta_{o}-BX_{d}\beta_{d}-{BX}_{w}\beta_{w}-\beta_{c}\right)}^{T}(ABy-BX_{o}\beta_{o}-BX_{d}\beta_{d}-BX_{w}\beta_{w}-\beta_{c})$$ (9)

In this paper, the maximum likelihood estimation is coded and processed in MATLAB, and two sets of estimation experiments have been conducted to show the code can recover the values of coefficients. Each experiment assumes 50 traffic analysis zones, implying a 50×50 = 2,500 observations in the simulated dataset. The primary difference between the two experiments lies in the true values of parameters, which allows for a more nuanced analysis of the model’s performance under varying conditions. Table 1 reports the true values, estimated values, t-statistics, and root mean squared errors (RMSE) of both experiments. Results indicate the MATLAB can achieve a good estimation of all coefficients in the SARAR model. In addition, the experiments demonstrate when spatial coefficients of origin and destination have different signs, and when spatial coefficients of the dependent variable and the error terms have different signs, MATLAB codes can achieve accurate estimation.

**Table 1:** Estimation results based on simulation data.

	Experiment 1				Experiment 2			
	True Values	Estimated Values	t-stat	RMSE	True Values	Estimated Values	t-stat	RMSE
***ρ***_***o***_	0.6	0.66	-3.03	0.0034	0.2	0.18	7.52	0.0003
***ρ***_***d***_	-0.4	-0.41	7.39	0.0002	0.2	0.17	4.83	0.0007
***ρ***_***w***_	-0.24	0.27	-	0.2625	-0.04	-0.03	-	0.0001
**λ**_***o***_	-0.5	-0.64	3.58	0.0200	-0.8	-0.79	-7.37	0.0001
**λ**_***d***_	0.5	0.47	-1.82	0.0011	-0.8	-0.70	-27.83	0.0101
**λ**_***w***_	-0.25	0.30	-	0.3020	-0.64	-0.55	-	0.0078
***β***_***c***_	2	1.95	4.33	0.0022	2	2.10	21.19	0.0110
***β***_***o***_	-2	-2.02	-3.97	0.0004	-2	-2.07	-32.43	0.0043
***β***_***d***_	1	0.85	9.80	0.0234	1	1.03	39.78	0.0008
***β***_***w***_	0.5	0.50	12.41	0.0000	0.5	0.51	17.86	0.0000
**Number of zones**	50				50			

The collection process and the analysis method complied with the terms and conditions for the source of the data.

## 4. Empirical study

### Data description

This paper uses a trip distribution dataset collected in Hangzhou, China, which comprises the traffic flow between 51 traffic analysis zones in the urban area, and a set of potential explanatory variables including population, key facilities, accessibility, and travel time (S1 Dataset). Specifically, this trip distribution dataset collects information from

- cellular signaling data. The cellular signaling data is provided by the China Mobile Communication Corporation, which is one of the three main cellular service providers in China and currently serves 69.56% of residents in Hangzhou. Though data does not correspond to the entire population, it can still reasonably represent the trip distribution pattern for the analyzed region. The data contains more than 30 million of records collected from 56 thousand base stations. Based on the time a user sending or receiving cellular signal, travelers’ physical movements are derived at the zonal level. Aggregating all users’ movement information enables the final trip distribution data. Details of deriving trip distribution data from raw data can be found in Ni et al. [6]
- transportation data. The transportation data is provided by local agencies of transportation construction and management. The data contains population, commercial area, and medical area for each traffic analysis zone. This paper leverages this transportation data to derive a number of explanatory variables to explain trip distribution.
- online map data. The online map data is provided by Baidu, one of the largest China’s technology companies. Their map service is similar to Google map but focusing on Chinese cities. The online map offers commercial buildings’ locations, road lane length, and travel time information.

Note the trip distribution data derived from the cellular signaling data are collected at different times: a Thursday morning (7am-10am with a total of 1,461,856 trips), a Thursday afternoon (4pm-7pm with a total of 1,436,328 trips), a Sunday morning (1,327,469 trips), and a Sunday afternoon (1,384,214 trips). This paper investigates trip distribution at all these times and compare their results. Table 2 is the description of all variables used in the SARAR model.

**Table 2:** Descriptive statistics of variables.

Variables	Description	Average	Standard Deviation	Min	Max
**Dependent Variables**					
Th_mor	Logarithm of traffic flow between the 51 traffic analysis zones on Thursday morning	-3.09	2.68	-9.21	4.47
Th_aft	Logarithm of traffic flow between the 51 traffic analysis zones on Thursday afternoon	-3.12	2.69	-9.21	4.44
Sun_mor	Logarithm of traffic flow between the 51 traffic analysis zones on Sunday morning	-2.82	2.12	-6.91	4.61
Sun_aft	Logarithm of traffic flow between the 51 traffic analysis zones on Sunday afternoon	-2.83	2.15	-6.91	4.63
**Explanatory Variables**					
Ln_O_pop	Logarithm of population of origin zones	0.90	1.40	-2.73	4.30
Ln_D_pop	Logarithm of population of destination zones				
O_comm	Sum of commercial centers of origin zones	1.08	1.49	0.00	7.00
D_comm	Sum of commercial centers of destination zones				
Ln_O_lane	Logarithm of road lane length in km over 100 of origin zones	0.57	0.83	-0.87	2.23
Ln_D_lane	Logarithm of road lane length in km over 100 of destination zones				
Transit	Number of transit lines between origin and destination	7.61	14.49	0.00	244.00
Time	Travel time between centroids of origin and destination in minutes	13.11	7.10	0.00	37.93

## 5. Results analysis

The SARAR model is used to investigate the trip distribution at different times. SAR and SEM models are also estimated for comparison purposes. The estimation results of the trip distribution on Thursday morning are reported in Table 3.

**Table 3:** Estimation results of Thursday morning.

	SARAR		SAR		SEM	
Variables	Coef.	t-stat	Coef.	t-stat	Coef.	t-stat
**rhoo**	0.67	9.20	0.86	62.11	-	-
**rhod**	0.68	8.75	0.88	70.61	-	-
**rhow**	-0.45	-	-0.75	-	-	-
**lambdao**	0.65	11.37	-	-	0.87	51.27
**lambdad**	0.68	4.93	-	-	0.88	62.07
**lambdaw**	-0.44	-	-	-	-0.76	-
**Ln_O_pop**	0.08	1.49	0.02	0.83	0.31	2.00
**Ln_D_pop**	0.10	1.07	0.03	1.50	0.37	2.56
**O_comm**	0.04	0.31	0.01	0.53	0.17	1.15
**D_comm**	0.06	1.45	0.02	1.03	0.21	1.53
**Ln_O_lane**	0.31	3.49	0.13	4.11	0.92	4.21
**Ln_D_lane**	0.26	2.12	0.12	3.67	0.74	5.10
**Transit**	0.02	1.67	0.02	8.59	0.02	8.91
**Time**	-0.02	-0.62	-0.02	-4.71	-0.02	-3.64
**constant**	-0.71	-4.47	-0.13	-1.79	-4.52	-2.74
**Log Likelihood**	-4.32E+03		-4.48E+03		-4.48E+03	
**AIC**	8.66E+03		8.98E+03		8.98E+03	
**BIC**	8.67E+03		8.98E+03		8.98E+03	

### Spatial coefficients

The spatial coefficients in SAR, ***ρ***{*ρ*_*o*_,*ρ*_*d*_,*ρ*_*w*_}, are 0.86, 0.88, and -0.75, respectively. They capture spatial autocorrelations at origin and destination, as well as the effect of their interaction term. The values of spatial coefficients are close to 1, indicating strong spatial dependences in the trip distribution data, and capturing spatial effects is necessary in establishing trip distribution models.

The spatial coefficients in SEM, ***λ***{*λ*_*o*_,*λ*_*d*_,*λ*_*w*_}, are 0.87, 0.88, and -0.76, respectively. From the model fitting perspective, both SAR and SEM have achieved satisfactory performance. The only difference is that the presence of spatial dependences is assumed to be in different mathematical terms: one assumes the spatial dependence take place on traffic flow, and the other assumes the spatial dependences takes place in the disturbance term.

Given spatial coefficients of both spatial autoregressive and spatial error terms are statistically significant, estimating SARAR model is the natural next step. Being able to specify spatial dependences on traffic flow and its disturbance term simultaneously, SARAR can disentangle and distribute spatial dependences across SAR and SEM. As shown in the log-likelihood values, SARAR’s value is greater than the log-likelihood values of SAR and SEM. In addition, Akaike Information Criterion (AIC) and Bayesian Information Criterion (BIC) show SARAR outperforms the alternative models. This is expected because all spatial coefficients are statistically significant in SARAR, adding explanatory power to the model. The values of spatial coefficients in SARAR are smaller than those in SAR and SEM. This is an interesting and important observation because

- Looking at spatial coefficients in the autocorrelation term alone in the SARAR model, its magnitude is smaller than the one in the SAR model. Such a difference indicates SAR tends to over-estimate the spatial effects, leading to higher marginal effects given both origin and destination spatial coefficients are positive.
- Similarly, the spatial coefficients in the error term alone in the SARAR model is smaller than the one in the SEM. Although the marginal effects do not involve spatial error term, the higher spatial coefficient values in SEM would jeopardize the effect of explanatory variables.
- Both ***ρ***{*ρ*_*o*_,*ρ*_*d*_,*ρ*_*w*_} and ***λ***{*λ*_*o*_,*λ*_*d*_,*λ*_*w*_} are smaller in SARAR implies the spatial effects in SAR and SEM are distributed across the spatial autoregressive term and the spatial error term.

### Marginal effects

Table 3 reports that most explanatory variables are statistically significant, though some of them have varying significances across models. In general, the SARAR model turns out to report the lower statistic significance, because the effect of explanatory is diluted due to the existence of more spatial coefficients. In other words, the true effect of explanatory variables should be understood coupled with the spatial effects. Specifically, the change in an explanatory variable would have an impact on flows out of the region (origin effect) and coming to the region (destination effect). When adding the spatial spillover effects onto the spatial interaction model, the marginal effects become even more complicated: both the origin effect and the destination effect will affect neighboring regions. To obtain the marginal effects, the following mathematical calculation has been conducted.

For models with spatial autoregressive terms, i.e., SARAR and SAR, the total marginal effects can be written in a matrix TE, which takes the form

$$TE=A^{-1}\left(\begin{array}{c} Jd_{1}\beta_{d}^{r}+Jo_{1}\beta_{o}^{r}\\ Jd_{2}\beta_{d}^{r}+Jo_{2}\beta_{o}^{r}\\ \vdots \\ Jd_{n}\beta_{d}^{r}+Jo_{n}\beta_{o}^{r} \end{array}\right)$$ (10)

where *Jd*_1_ and *Jo*_1_ are a *n*×*n* matrix with the affected regions equal to 1. Multiplying them with $\beta_{d}^{r}$ and $\beta_{o}^{r}$ shows total effects of the *r*-th explanatory variables on the travel flow. Then, a scalar summary measure of the total effect of changes in the *r*-th explanatory variable can take the form

$$te=\frac{1}{n^{2}}∙\iota_{n^{2}}\prime ∙TE∙\iota_{n}$$ (11)

Additionally, the derivation of the origin effect (OE), destination effect (DE), intraregional effect (IE) and network effect follows a similar rationale. For detailed explanation, please refer to LeSage and Thomas-Agnan [3].

This paper has calculated the marginal effects (total effect, origin effect, destination effect, intraregional effect, and network effect) for the trip distribution on Thursday morning, Sunday morning, Thursday afternoon, and Sunday afternoon. The estimation results are reported in Table 4. The interpretation becomes straightforward. For example, the results of population on Thursday morning by the SARAR model imply that for every 1% increase in population, the total number of trip would increase by 1.77%. This 1.77% is constituted by the origin effect (0.73%), destination effect (0.94%), intraregional effect (0.04%), and network effect (0.07%). In other words, the decomposition of marginal effects reveals how the effect of explanatory variables would take place in the spatial processes.

**Table 4:** Estimated spatial coefficients and marginal effects for all models.

	Thursday Morning			Sunday Morning			Thursday Afternoon			Sunday Afternoon		
Spatial Coefficients	SAR	SEM	SARAR	SAR	SEM	SARAR	SAR	SEM	SARAR	SAR	SEM	SARAR
***ρ***_***o***_	0.86***	-	0.67***	0.81***	-	0.62***	0.86***	-	0.67***	0.81***	-	0.63***
***ρ***_***d***_	0.88***	-	0.68***	0.83***	-	0.63***	0.88***	-	0.68***	0.83***	-	0.64***
***ρ***_***w***_	-0.75	-	**-0.45**	-0.67	-	**-0.39**	-0.76	-	**-0.45**	-0.67	-	**-0.40**
***λ***_***o***_	-	0.87***	0.65***	-	0.83***	0.59***	-	0.87***	0.65***	-	0.83***	0.60***
***λ***_***d***_	-	0.88***	0.68***	-	0.84***	0.61***	-	0.88***	0.68***	-	0.84***	0.62***
***λ***_***w***_	-	-0.76	**-0.44**	-	-0.69	**-0.36**	-	-0.76	**-0.44**	-	-0.69	**-0.37**
Population (ln)	SAR	SEM	**SARAR**	SAR	SEM	**SARAR**	SAR	SEM	**SARAR**	SAR	SEM	**SARAR**
Total Effects	2.78	0.68	**1.77**	2.66	0.76	**1.71**	2.67	0.68	**1.73**	2.44	0.73	**1.66**
Origin Effects	0.86	0.29	**0.73**	1.14	0.36	**0.79**	0.70	0.27	**0.65**	0.94	0.32	**0.70**
Destination Effects	1.76	0.35	**0.94**	1.36	0.36	**0.82**	1.81	0.37	**0.98**	1.36	0.37	**0.87**
Intraregional Effects	0.06	0.01	**0.04**	0.05	0.02	**0.03**	0.05	0.01	**0.03**	0.05	0.01	**0.03**
Network Effects	0.11	0.03	**0.07**	0.11	0.03	**0.07**	0.11	0.03	**0.07**	0.10	0.03	**0.07**
Commerce	SAR	SEM	**SARAR**	SAR	SEM	**SARAR**	SAR	SEM	**SARAR**	SAR	SEM	**SARAR**
Total Effects	1.59	0.39	**0.98**	1.03	0.32	**0.72**	1.61	0.39	**0.98**	1.12	0.34	**0.79**
Origin Effects	0.47	0.16	**0.37**	0.30	0.13	**0.27**	0.56	0.17	**0.41**	0.40	0.15	**0.32**
Destination Effects	1.03	0.20	**0.54**	0.67	0.17	**0.41**	0.96	0.19	**0.51**	0.65	0.18	**0.42**
Intraregional Effects	0.03	0.01	**0.02**	0.02	0.01	**0.01**	0.03	0.01	**0.02**	0.02	0.01	**0.02**
Network Effects	0.06	0.02	**0.04**	0.04	0.01	**0.03**	0.06	0.02	**0.04**	0.04	0.01	**0.03**
Transportation Lanes (ln)	SAR	SEM	**SARAR**	SAR	SEM	**SARAR**	SAR	SEM	**SARAR**	SAR	SEM	**SARAR**
Total Effects	14.68	1.66	**5.36**	6.77	1.01	**3.01**	15.16	1.71	**5.55**	7.80	1.17	**3.58**
Origin Effects	7.04	0.87	**2.69**	3.10	0.49	**1.42**	7.43	0.90	**2.84**	3.74	0.60	**1.76**
Destination Effects	6.77	0.70	**2.35**	3.27	0.46	**1.41**	6.82	0.71	**2.38**	3.60	0.51	**1.61**
Intraregional Effects	0.29	0.03	**0.11**	0.13	0.02	**0.06**	0.30	0.03	**0.11**	0.16	0.02	**0.07**
Network Effects	0.59	0.07	**0.21**	0.27	0.04	**0.12**	0.61	0.07	**0.22**	0.31	0.05	**0.14**

+ p<0.1

* p<0.05

** p<0.01

*** p<0.005.

Comparing the marginal effects among SAR, SEM, and SARAR models, SAR models turn out to have the largest values. The dominant reason is that the spatial coefficients are close to 1 so that the spatial filtering enlarges the effect of explanatory variables. On the other hand, SEM reports the smallest marginal effects. The values of SAR models are in between, which is a direct response to the paper’s hypothesis that SARAR specifications can balances the marginal effects obtained from SAR and SEM due to the distributed spatial effects at the dependent variable and the error term.

### Effects of day-in-week and time-of-day

Comparing Thursday morning with Sunday morning, marginal effects of commerce and the number of road lanes are vastly different. The difference has accounted for the difference in total number of trips–the total number of trips on Thursday morning is about 10% higher than Sunday morning. The total effect of commerce on Thursday is 0.98, indicating for every 1 unit difference in the number of commercial facility, the total number of trip would increase by 0.98%. The total marginal effect of commerce on Thursday is about 35% higher than Sunday, indicating commercial facilities produce 35% more trips on weekdays than weekends. Regarding road lane length, the difference is even greater. The total effect on Thursday morning is about 78% greater than weekend. This finding is quite intuitive as traffic congestion is often much worse on weekdays, and willingness to travel is sensitive to additional road capacity.

A Similar finding of the Thursday and Sunday difference can be found during afternoon hours. Commerce is 24% higher and road length is 50% higher on Thursday. The difference in marginal effects implies trip distribution modeling should be conducted for different day-in-week. This paper provides empirical estimation of marginal effects by the SARAR model.

Comparing afternoon’s results against morning results, all differences are less than 10% and most are less than 5%. This finding implies shows the relationship between explanatory variables and trip distribution is consistent over morning hours and afternoon hours. If the transport modeling resource is limited, analysts are not recommended to run spatial interaction models separately for mornings and afternoons.

### Implications for Hangzhou 2035

Given that the SARAR model has been specified and estimated, the next step is to investigate how it may be applied to analyze empirical trip distribution data. Based on the estimation result on the Thursday’s morning, and a special development plan proposed by the Hangzhou municipal government, the prediction of trip distribution with respect to longer road lanes can be obtained. In other words, this empirical analysis serves as an example for practitioners to use the SARAR model to make reasonable trip distribution predictions. Generally speaking, the application of SARAR models is indifferent to the traditional trip distribution analyses in terms of determining zones, explanatory variables, and making predictions. The only difference is in specifying the trip distribution models and deriving the marginal effects.

In September 2020, the municipal government of Hangzhou announced the Special Plan for Comprehensive Transportation of Hangzhou (2021–2035). One of the key objectives is to promote a green and intelligent transportation system. The whole city is divided into three types of zones: the demonstration development zones, the priority development zones, and the guidance development zones. The demonstration zones and the priority zones aim to have an increased road lane density, which are more than 10km/km2 and 8 km/km2, respectively. Traffic analysis zones 26,38,43,45,48,49 and 50 represent development zones the corresponding zones road lane length increases are shown in Table 5.

**Table 5:** Current and projected road lane length.

Traffic Analysis Zone Number	2020 Road Lane Length (km)	2035 Road Lane Length (km)	Percentage of Increase
26	145.2	181.3	24.9%
38	738.5	917.8	24.3%
43	324.3	393.6	21.4%
45	697.4	1206.3	73.0%
48	513.4	542.4	5.6%
49	529.2	640.7	21.1%
50	344.8	655.1	90.0%

As the length of road lanes is an important influential factor of trip distribution, holding other explanatory variables constant, the projected increase of length will lead to higher traffic volume from and to these zones. With the SARAR specification assumption, the mechanism of traffic volume increase consists of origin effects, destination effects, interregional effects, and network effects. Table 6 reports the scalar summary of these effects for Thursday morning’s travel patterns. Traffic volume increases on the other times can be derived following the same procedure.

**Table 6:** Projected average traffic volume increase due to longer road lanes at certain zones with the SARAR specification.

Traffic Analysis Zone Number	Total Effect	Origin Effect	Destination Effect	Intraregional Effect	Network Effect
26	2.32%	1.20%	0.99%	0.04%	0.09%
38	2.23%	1.17%	0.94%	0.04%	0.08%
43	2.00%	1.05%	0.84%	0.04%	0.08%
45	5.61%	2.96%	2.34%	0.11%	0.21%
48	0.57%	0.30%	0.24%	0.01%	0.02%
49	2.01%	1.03%	0.87%	0.04%	0.08%
50	6.72%	3.46%	2.87%	0.13%	0.26%

Due to the increased lane length at zone 26, the traffic volume from and to zone 26 will increase. Moreover, zones around zone 26 will observe an increase with the effect of spatial dependences. In terms of the magnitude of effects, based on the calculation of TE matrix, zone 26 itself is increasing by the greatest percentage, and the effects diminishes as the distances to zone 26 are increasing.

Note that due the differences in model specification, SARAR, SAR and SEM models will observe different projections in traffic volume increase given the same lane length increase assumption. In this particular empirical study, SAR would offer the greatest traffic volume increase, and followed by SARAR. SEM would result in the lowest traffic volume increase. In terms of selection of best project, this paper recommends SARAR as it has the best goodness-of-fit.

## 6. Conclusions and discussions

This paper specifies, estimates, and applies the spatial autoregressive model with spatial autoregressive disturbances with OD filterings to investigate trip distributions. Apart from being an early work of adopting new models to study a traditional problem, this paper also contributes to the existing literature by (1) filling the void that no existing studies have examined the difference in the spatial OD dependence specification, and how different specifications drive the variation in marginal effects of explanatory variables, and (2) comparing the empirical marginal effects across mornings and afternoons on the weekday and weekend with empirical data.

In general, the empirical use of spatial interaction models with spatial OD filterings is far from comprehensive in modeling trip distributions. In the era with big data in the transportation industry, the need of adopting the OD filtering models to improve the understanding of travel patterns is increasing. Hence, this paper has proven that the trip distribution study is a great use case for the proposed OD filtering models. Continuing this line of research has both academic and practice values in the transportation planning and management.

From the theoretical and empirical analyses, this paper concludes that 1) the spatial process in the trip distribution data takes place in both the SAR and SEM terms. 2) SARAR models can investigate the SAR and SEM simultaneously and the estimated spatial coefficients can reveal the intensity of each spatial effect. 3) SARAR models outperform the SAR and SEM models in all investigated scenarios so that the marginal effects obtained from SARAR should be the best to use in practice from the statistical point of view. 4) Thursday and Sunday present different marginal effects for the commerce and road lane length variables, while morning and afternoon do not exhibit major differences.

The limitations of this study are 1) investigating trip distribution at the same time on two different days may introduce the panel data specifications. Researchers have introduced the fixed effects model [14] but is not yet suited for this paper, because the explanatory variables constant over time would be canceled out in estimation. 2) Data collection of explanatory variables indexed by separate times is expected to examine policy interventions of trip distribution. Hence, a foreseeable next step is to develop time-varying spatial OD dependence models and collect more comprehensive trip distribution data.

## Supporting information

S1 Dataset Analysis data submitted. (XLSX)
